# Protein Supplement Perceptions, Use, and Associated Performance in Young Lebanese Resistance-Training Athletes

**DOI:** 10.1155/2022/4150620

**Published:** 2022-02-18

**Authors:** Khadije K. Saleh, Sofi G. Julien

**Affiliations:** Department of Human Nutrition and Food Sciences, Faculty of Arts Humanities and Sciences, Holy Spirit University of Kaslik, P.O. Box 446, Jounieh, Mount Lebanon, Lebanon

## Abstract

The aims of this study were first to evaluate the nutritional knowledge, perception, and source of nutrition information among resistance-trained individuals consuming protein supplements (PS), to determine whether a correlation exists between nutrition-related knowledge and the use of PS, and finally to compare the impact of PS use among participants classified as nonprotein supplement users (NPSUs) and protein supplement users (PSUs). A cross-sectional study was conducted among a highly selected group of resistance-specialized trainees (RSTs). Among the 100 RST participants recruited, the Internet and coaches were the most common source of nutritional information. About one-third of participants believed that there were no health risks after consuming PS. Both NPSU and PSU exhibit performance improvement that was significantly lessened in PSU compared to NPSU. This study demonstrated that RST may have misconceptions regarding the benefits of PS usage to increase strength. Our data also suggest a shortage of knowledge about PS and confirm that PSUs lack proper professional guidance. These findings highlight the need for proper monitoring to ensure adequate perception, awareness, and safety in the Lebanese sports sector.

## 1. Introduction

Protein supplements are among the most commonly used dietary supplements and have been widespread in the general population from recreational to well-trained exercisers to enhance health and athletic performance. The misconception that a product is safe simply because it is marketed over the counter became a severe health concern worldwide. There are health risks associated with the consumption of dietary supplements. The health benefits of these agents are unclear and there is no reliable data to support their widespread use [1]. However, supplements are nowadays commonly considered as food and therefore extensively consumed despite a limited knowledge around their efficacy, safety, and regulatory policies, unlike their drug counterparts [2]. Although dietary supplements and other nutrition products represent a legal business strategy, a recent research affirms that exceeding daily nutrient requirements lacks efficacy and may be associated with potential toxicity. Most of these supplements are used in a chaotic way in relation to their quantity, frequency, and duration [4, 5]. More than a decade ago, the use of supplements was questionable, and unrefined food was considered to be a better asset over dietary supplements due to the presence of certain factors in whole food known to have greater importance than the level of individual nutrients ingested in their pill-or powder forms [6, 7]. Efficacy studies of various performance-enhancing substances are regularly published, and outcomes are often equivocal, making the use of the substance controversial and confusing [8–10].

Manufacturers often use deficiency models or animal studies and generalize the results to a healthy human population. Poor research design or inappropriate extrapolation of results should be recognized as poor scientific evidence, but with the development of social media and free-access information, dietary consumers are now exposed to misinformation and risk in terms of the safety and efficacy of food supplements [11–13]. It has been shown that trainees relied more on Internet, friends, or coaches than professionals to get nutrition-related information as reviewed by Denham [14]. Ironically, gym instructors and coaches are insufficiently trained to provide accurate information about the use of food supplements, and qualified health coaches or dietitians are becoming increasingly ignored in gyms in Lebanon [15].

Several studies have demonstrated that resistance training has major effects on muscular strength and hypertrophy [16] while other works also indicated that resistance exercise stimulated the synthesis of muscle proteins which is further stimulated by protein intake as recently reviewed by McKendry et al. [17]. Recent evidence supports the benefits of resistance training in increasing and/or maintaining lean body mass and bone mineral density [18, 19] and highlights their effects on muscular strength and hypertrophy [20, 21]. While Phillips reviewed the relative merits of higher protein diets for athletes and argued that it would come at the expense of another macronutrient, others comforted his conclusion that exercise-induced muscle mass would be controlled by the balance between muscle protein synthesis and breakdown [22] or that resistance exercise stimulated the synthesis of muscle protein, which was further stimulated by protein intake, encouraging athletes, therefore, to combine resistance training with protein supplementation [16].

A growing body of literature agrees that an adequate protein intake for strength training athletes should range between 1.2 and 1.7 g/kg/day, a limit beyond which any protein supplementation failed to augment resistance exercise-induced muscle hypertrophy or muscle growth [23, 24]. Recently, it was suggested that the protein intake required to counterbalance the reductions in lean body mass is likely dependent on the severity of energy restriction and the amount of resistance exercise habitually performed [25]. Several studies performed by the group of Philip and others showed that protein supplementation did not further increase muscle strength among individuals who consumed adequate amounts of dietary protein [24, 26, 27]. However, with the aim of maximizing performance, individuals seeking to gain muscle mass are likely to consume more protein with the misconceived belief that large quantities of protein consumption might generate more muscle protein [28]. The supplementation prevalence level was found to be remarkably high in Lebanon and even excessive [29] despite the fact that, using supplements, users were reported to experience one or several side effects [30] and to lack awareness on the use and safety of dietary supplements [15].

Altogether, this data highlight malpractice and inappropriate intake of food supplements in athletes worldwide while yet poorly documented in resistance-trained Lebanese individuals. Also, because those resistance-specialized trainees (RSTs) typically use protein supplementation for its speculated positive effects on enhancing gains in muscle mass and promotion of lean mass retention [15, 29, 30], the aims of this study were to, first, evaluate nutrition knowledge and the habits of protein supplementation among strength practitioners and, second, to correlate the protein supplementation practices with the performance of resistance-trained individuals. Analyzing those correlations would present novel evidence for more comprehensive nutrition services in regard to the serious public health concerns about the chaotic PS usage in Lebanon.

## 2. Materials and Methods

### 2.1. Study Design and Participants

A repeated cross-sectional study was performed with 100 resistance-trained nonelite athletes (RTAs) in Beirut, Lebanon. Due to lockdown restriction measures taken in Lebanon during the COVID-19 pandemic, additional recruitment of individuals was no longer possible as gym facilities remained closed, which explains why the study was restricted to 100 eligible participants. Exclusion criteria for all participants were (1) age below 20 and above 40, (2) nonresistance-specialized trainee, and (3) use of steroids or corticoids. Inclusion criteria were as follows: (1) all participants must be exclusively resistance-specialized trainees, (2) there should be strength-specific and 1 repetition-maximum (1RM) load program in gyms facilities, and (3) there should be exclusive membership to one of the resistance-training facilities. Three gyms met those criteria: Barbel House, Crossfit B-Town, and Crossfit 961.

This study was approved by the Ethics Committee of the Holy Spirit University of Kaslik (USEK). Before collecting data, an informed written consent was signed by all volunteer participants. A comprehensive questionnaire, the nutrition for sports knowledge questionnaire (NSKQ) [31], made of 54 questions was designed to collect information based on numerous factors related to (1) demographics, including age, education level, disease history, smoking, and alcohol status; (2) exercise behaviors, including type, frequency, and total time of exercise and aim of exercising; (3) lifestyle habits, including the type of diet, daily caloric intake, frequency of supplement intake, and food protein intake; (4) source of nutrition information and awareness of the risk of any supplement use; (5) performance for 1RM in back squat, 1RM in the bench press, 1RM in the deadlift and set of total pull-ups; and finally, (6) nutrition-related knowledge including 6 subcategories comprising carbohydrates, protein, fat, vitamin, and general and advanced knowledge. Questionnaires were distributed to the participants for a face-to-face interview in the gyms before training sessions during weekdays. Participants were divided into two main groups according to their declaration to use protein supplements or not. Nonprotein supplement users (NPSUs) group (*n* = 51) was systematically compared to the Protein Supplement User (PSU) group (*n* = 49) for questionnaire analyses. Participants were instructed to keep the same frequency during the length of the study to ensure consistent results.

### 2.2. Performance Outcomes

Performance was assessed in two different interviews 8 weeks apart for all the participants. More specifically, upon introducing the study objectives and requirements, the study interviewer agreed with all participants to select a date that was considered week 0 in which the interviewer was personally present at the time of filling the questionnaire and when each participant performed individual 1RM, which has been reported to safely and efficaciously evaluate muscular strength [32]. On week 0, the study personnel recorded the 1RM of each participant's back squat, 1RM bench press, 1RM deadlift, and set of pull-ups on each participant's questionnaire. This was repeated under the supervision of the same interviewer 8 weeks after the initial recording. All programs that were followed by the participants (whether taking protein supplements or not) were strength-specific and train at a specific repetition-maximum load. Most of the participants (whether taking protein supplements or not) have been exercising for more than 1 year.

### 2.3. Knowledge Scoring

The total overall score for knowledge was calculated for each participant as the total number of correct answers and was divided into 3 groups as poor (score <20), satisfactory (score ranging from 20 to 39), and high (score >40). For an in-depth analysis of knowledge answer outcomes, each of the 6 subcategories consisting of a total of 29 questions with a total of 53 items was computed into different scores as per the total number of questions.

### 2.4. Statistical Analysis

All collected data were computed and analyzed using SPSS Version 22 (IBM Corporation, Chicago, USA) for all categories of the questionnaire except the performance that was analyzed using GraphPad Prism 9 (GraphPad Software Inc., San Diego, USA). All descriptive analyses were based on frequencies and percentages. Pearson's *χ*^2^ square test was performed for all question analyses, and a Fisher's exact test was used instead when the expected values < 5 were more than 20%. All value data were presented as mean ± Standard Deviation (SD). The *P* value was considered significant when *P* < 0.05. Cramer's V test was used to comfort the strength of correlation between knowledge and protein supplement intake and was considered moderate when *ϕ* ranged between [0.3 and 0.7] and strong when *ϕ* > 0.7.

## 3. Results

### 3.1. Prevalence of Demographics and Lifestyle Characteristics of Dietary Supplement Users

Demographics of the hundred participants categorized into nonsupplement users (38%) and supplement users (62%) are shown in Supp.  Table 1. Most of the subjects were male, and all were mostly young between 20 and 30 years of age and declared earning a university degree. A low prevalence of disease history among participants was observed. Participants were mostly nonsmokers in both categories, with a higher percentage of nonsmokers in supplement users while most of them admitted alcohol consumption at least once weekly.

Subsequently, the supplement users were subdivided into two categories as nonprotein supplement users (NPSUs) and protein supplement users (PSU) to assess a potential relationship between demographics and lifestyle characteristics and the type of dietary supplementation. As shown in Supp.  Table 2, there was no significant difference when the supplement consumption was compared among all demographic criteria. Likewise, the use of protein supplements was not significantly associated with age, education level, disease history, smoking status, and alcohol intake. Among all dietary supplement users, a great majority of NPSU (80,4%) and PSU (81,6%) reported eating a balanced diet. Strikingly, most of them were not aware of their caloric intake and the quantity of food protein consumed daily (Supp.  Table 2).

### 3.2. Nutritional Knowledge Rating, Nutrition Information Sources, and Awareness of Health Risks

When assessing nutrition-related knowledge using the nutrition for sports knowledge questionnaire (NSKQ) [31], the majority of both NPSU (84.3%) and PSU (79.6%) ranked among the satisfactory knowledge score of 20–39 out of a total score of 54, as shown in Table 1.

An in-depth analysis of the NSKQ (Supp.  Figure 1) showed intriguing results concerning general knowledge since only half (55%) of participants answered correctly on the average fluid needed per day, and only 9% answered correctly on the percentage of body dehydration that would induce a decrease in exercise performance (Supp.  Figure 1; Q46-47, respectively). Fifty-three percent of participants disagreed that a high protein meal is recommended 1 hour before the competition (Supp.  Figure 1; Q48), 79% correctly answered that a high carbohydrate meal 2–4 hours before exercise can lead to improvements in endurance performance (Supp.  Figure 1; Q49), and 42% agreed that it is best to consume high glycemic index carbohydrates after exercise to support muscle glycogen recovery (Supp.  Figure 1; Q53). Furthermore, only 30% of the participants knew that fat is the most energy-dense macronutrient with 9 kcal/g (Supp.  Figure 1; Q45c) while about half of them answered unsure in the amount of energy per gram for macronutrients (Supp.  Figure 1; Q45a-d). About 19% answered that carbohydrates should be the main source of energy and spare protein (Supp.  Figure 1; Q27 and Q29, respectively). However, 78% of the study participants were aware that protein's main use is for growth and repair (Supp.  Figure 1; Q31). An overall 68% of participants correctly answered questions on fat sources (Supp.  Figure 1; Q40a-h), and only 55% knew about saturated and unsaturated fat recommendation (Supp.  Figure 1; Q39) whereas the majority of them were able to identify foods that were high or low in carbohydrates (Supp.  Figure 1; Q26a-g) and high or low in protein (Supp.  Figure 1; Q30a-i). About the energy requirement, only 30% of participants knew what proportion of energy should come from protein (Supp.  Figure 1; Q37), and when asked about carbohydrates, 20% selected a value below the current recommendations (Supp.  Figure 1; Q27). Finally, the mean score of vitamin-related knowledge was 40.3% as shown in Table 1. Half of the participants were aware of the role of antioxidants in the body (Supp.  Figure 1, Q41) while only a quarter knew why B vitamins are important for exercise (Supp.  Figure 1 Q42).

Finally, we did not observe any significant association between PSU and nutritional knowledge score (*P* = 0.733) expected for advanced knowledge, related to sports nutrition, which was scored higher for PSU than for NPSU (30.1% *vs* 18.7%, respectively) as shown in Table 1. Altogether, our data indicated that PSU displayed a significant difference only in the advanced knowledge category compared with NPSU (*P* < 0.0001) with moderate correlation (*ϕ* = 0.394) (Table 2).

As shown in Table 1, the Internet and sports coaches were the highest reported sources of information whereas 8% only reported consulting a dietitian. The source of nutrition-related information was not significantly associated with the type of dietary supplement. The use of the Internet, however, was more prevalent as the main source of information for NPSU, while sports coach was the main reference for PSU instead.

Surprisingly, a large proportion of NPSU and PSU believed that there are no health risks associated with protein supplementation (47.1% and 34.7%, respectively) while about the other half of the dietary supplement users believed that the health risk associated with protein supplementation was the harm of kidney function as shown in Supplemental Table 3.

### 3.3. Supplement Use

As previously shown in Supplementary Table 1, men displayed a higher prevalence (66.7%) than women (54.1%) as dietary supplements users among all participants. About 80% of the dietary supplement users reported consuming whey protein as a protein supplement with a higher prevalence among men than women, and the frequency of protein supplement intake was predominantly 5–6 times per week for men and 3 times per week for women (data not shown).

### 3.4. Exercises Behavior Associated with Protein Supplement Usage

A descriptive analysis of the supplement usage showed that the longer the participants have been training, the more important the prevalence of supplement usage was among the PSU (Table 3), and more than half have been training for more than a year at a frequency of more than 5 times per week from 1 to 2 hours a day. Protein supplement usage appeared to be the highest among participants who have been practicing more than three times a week and more than 1 hour a day. The use of protein supplements was moderately correlated with the total time of daily exercise (*ϕ* = 0.318; *P* = 0.006).

### 3.5. Performance

The effect of individual performance illustrated in Supp. Figures 2A-D for 1RM back squat, 1RM bench press, 1RM deadlift, and set of pull, respectively, of protein supplementation was examined in PSU (*n* = 49) and NPSU (*n* = 51). For in-depth analysis, the performance was also inspected among the protein supplement users and those that did not use any kind of supplements (*n* = 38) in order to avoid biases related to the effect that may result from the usage of other types of dietary supplement on performance. An increase for all performance exercises in both NPSUs and PSUs was observed as shown in the upper part of Table 4. Interestingly, the increase in performance observed at week 8 was more prominent in NPSUs than PSUs. Indeed, as shown in the lower part of Table 4, the percentage change in the back squat was about 9% in NPSUs whereas PSU change was only 4%. The same applied to bench press (8% *vs* 3%), deadlift (11% *vs* 5%), and pull-ups (14% *vs* 9%). Moreover, all the mean differences for performance improvement between week 0 and week 8 were significant (*P* < 0.0001) in both NPSUs and PSUs (Table 4). The mean increase (±SD) for the four kinds of exercise was significantly lower in the PSU at week 0 than at week 8 as compared to NPSU during the same time period.

## 4. Discussion

The aim of this study was first to assess the nutritional knowledge and use of protein supplements in a population of resistance-trained individuals in the town of Beirut, Lebanon, and second to characterize the type of correlation between protein supplement consumption and exercise performance. Previous studies conducted in Lebanon in 2012 reported a prevalence of 36.3% for the consumption of dietary supplements [29], which increased to 49.5% in 2016 [15], and reached now 62% as demonstrated in our study. From those two previous studies, we also confirmed that dietary supplementation was more prevalent among males than females. However, we did not observe any correlation between protein supplementation with gender, age group, level of education, disease history, smoking status, and alcohol intake. Our finding that males are more likely to consume protein supplements might be attributed to the fact that males were exercising more frequently than females as previously reported [33].

Remarkably, although the majority of the PSU in this study reported having a balanced diet, more than a quarter did not know how many calories and grams of food protein they were consuming. Our data raised an important issue recently highlighted by Salami et al. They reported that while carbohydrate and fat intakes were below the RDA in the majority of users and nonusers of dietary supplements, in contrast, the protein intake of most of the gym users exceeded their needs [30]. This might be explained by the observed unawareness by PSU and NPSU of energy requirements in terms of calories per gram of macronutrients [34].

We also observed that protein supplement use increased among participants with long habits of exercising and that most protein supplement users were exercising for more than 1 year with a prevalence of 67.3%. Those with the highest frequency of resistance training like 3–5 times a week showed a higher prevalence of dietary supplement use compared to lower frequencies (less than 3 times a week). Although our observations are in accordance with other Lebanese studies [15, 29], there were however no statistically significant differences between frequency categories. A higher number of participants might be necessary to evidence a correlation between those two variables.

In previous studies, researchers have raised concerns regarding the validity of the current nutrition knowledge measures among exercisers [35–37]. Given the large degree of discrepancy in the format and type of the questions across measures, scores reported might provide little information regarding the actual knowledge and the gaps in knowledge among the participants. Therefore, the measurement tool we used in this study questionnaire included all the subsections recommended by a recent systematic review [38]. Studies that compared nutrition subsections similar to ours found that scores were better in the sports nutrition knowledge compared to the general nutrition knowledge section [39, 40].

About the nutrition information sources, our study showed that resistance-trained individuals often use dietary supplements without scientific evidence. It appeared that while only a small proportion reported seeking professional advice from a dietitian, the vast majority of our participants relied mainly on the Internet and sports coaches for supplement indications. The observed frequencies were previously reported in several studies in Lebanon and worldwide where the highest percentage of supplement users were seeking information mainly from coaches and/or the Internet while only a lower proportion consulted a dietitian [15, 29, 35, 36, 39]. A recent study concluded that the Lebanese coaches and athletic trainers were not sufficiently ready to deliver quality information about sports nutrition and reported that only 26.5% of trainers have undergone formal nutrition training. They obtain sports nutrition information mainly via the Internet and magazines [41]. Furthermore, any additional cost to gym membership fees may discourage PSU to request a sports nutrition dietitian consultation. Despite the limited number of participants in our study related to the eligibility criteria of the selection of gym's facilities that targeted exclusively resistance trainees for more accuracy of data analysis, our results together with others from Lebanon and worldwide implied that sports coaches are not sufficiently qualified to deliver reliable information about sports nutrition.

Another aspect of nutrition knowledge deals with health risks associated with supplement consumption. Our findings corroborate with two studies where more than 60% of the participants claimed the absence of side effects related to the usage of protein supplements [15, 42]. Surprisingly, although more than half of our PSUs reported that protein supplementation harms kidney function, they still choose to consume it. These findings are in line with the study by Salami et al. in which supplement users reported having experienced one or more adverse events of supplementation including hypertension, polyuria, mood swings, and excess facial hair but still choose to consume it [30, 41].

With health professionals possessing the most appropriate evidence-based source of information on dietary supplements, it is of great concern that resistance-trained individuals continuously turn to other less reliable sources of information. Athletes receiving information from any source other than a health professional are at risk of getting misleading and hazardous information. Further investigations in the area of self-prescription and the reason for taking dietary supplements can be necessary to understand that growing trend.

Perhaps not surprisingly, the association of protein supplementation to improved performance can be attributed to the overwhelming marketing and advertisements that play a role in that false belief through the dissemination of nutrition-related information. In this study, significant increases in 1RM back squat, bench press, deadlift, and pull-up were observed in both NPSU and PSU after 8 weeks. Interestingly, the percentage change in performance measures was higher among nonsupplement users compared to supplement users. To our knowledge, our study is the first one in Lebanon that has investigated the supplementation effect on the performance of resistance-trained individuals. Interestingly, our data confirmed that recent clinical research showing physical activity level was not associated with protein supplement frequency [43] and also reflected the findings of Longland et al. in which all measures of exercise performance improved similarly in the protein and control groups as a result of the intense exercise intervention with no effect of protein supplementation [44].

## 5. Conclusion

To our knowledge, this is the first study in Lebanon that found an association between the nutrition-related knowledge of the participants and supplements usage. Our data indicate that protein supplement consumption in resistance-trained individuals during 8 weeks of resistance training does not provide any added benefit to participant performance. We have shown that our participants were unaware that individuals involved in intense training and with elevated dietary protein needs can achieve these recommendations with a well-balanced diet that maintains energy balance and that supplementation may only benefit individuals with a poor diet, preexisting deficiencies, or individuals who are on caloric restrictions. Therefore, our study raises the need for the significant role of health professionals such as health educators, general practitioners, and sports dietitians to help individuals who are considering purchasing or using dietary supplements assess the rationale for using such products.

## Figures and Tables

**Table 1:** Nutritional knowledge.

	NPSU *n* (%)	PSU *n* (%)	*P* value
*Knowledge score*			
Poor: <20	6 (11.8)	6 (12.2)	0.733
Satisfactory: 20–39	43 (84.3)	39 (79.6)	
High: ≥40	2 (3.9)	4 (8.2)	

*Knowledge*			Total %
Carbohydrate	28.6	28.5	57.1
Protein	29.5	31.8	61.3
Fat	33.8	34.5	68.3
Vitamins	18.5	21.8	40.3
General	15.5	17.1	32.6
Advanced	18.7	30.1	48.8

*P* value: Fisher's exact test.

**Table 2:** Nutritional knowledge associated with protein supplement usage.

	Number of questions	Range	NPSU, *n* (%)	PSU, *n* (%)	*P* value	Cramer V (*ϕ*)
Carbohydrate	9	<4: low	7 (13.7)	7 (14.3)	0.812	
		4–6: satisfactory	36 (70.6)	32 (65.3)		
		>6: high	8 (15.7)	10 (20.4)		
Protein	18	<8: low	10 (19.6)	7 (14.3)	0.089	
		8–12: satisfactory	25 (49)	16 (32.7)		
		>12: high	16 (31.4)	26 (53.1)		
Fat	9	<4: low	5 (9.8)	4 (8.2)	0.302	
		4–6: satisfactory	25 (49)	17 (34.7)		
		>6: high	21 (41.2)	28 (57.1)		
Vitamins and minerals	2	0: low	22 (43.1)	16 (32.7)	0.543	
		1: satisfactory	21 (41.2)	23 (46.9)		
		2: high	8 (15.7)	10 (20.4)		
General knowledge	8	<4: low	41 (80.4)	31 (63.3)	0.057	
		4–6: satisfactory	10 (35.7)	18 (36.7)		
		>6: high	0 (0.0)	0 (0.0)		
Advanced knowledge	7	<3: low	20 (39.2)	8 (16.3)	^*∗∗∗*^	0.394
		3–4: satisfactory	21 (41.2)	13 (26.5)		
		>4: high	10 (19.6)	28 (57.1)		

*P* value: Pearson's Chi-square test and Fisher's exact test with more than 20% of expected counts less than 5 (fat). ^*∗∗∗*^*P* < 0.0001.

**Table 3:** Exercise behavior associated with protein supplement usage.

	NPSU, *n* (%)	PSU, *n* (%)	*P* value	Cramer V (*ϕ*)
*Total time of exercise*				
<1 month	6 (75)	2 (25)	0.542	
1–6 months	11 (52.4)	10 (47.6)		
7 months–1 year	3 (50)	3 (50)		
>1 year	31 (47.7)	33 (52.3)		

*Frequency of exercise*				
<3 times/week	13 (68.4)	6 (31.6)	0.085	
3–5 times/week	31 (51.7)	29 (48.3)		
>5 times/week	7 (33.3)	14 (66.7)		

*Total time of daily exercise*				
<1 hour/day	16 (66.7)	8 (33.3)	^*∗∗*^	
1–2 hours/day	34 (52.3)	31 (47.7)		0.318
>2 hours/day	1 (9)	10 (91)		

*P* value: Pearson's Chi-square and Fisher's exact test with more than 20% of expected count less than 5 (total time of exercise). ^*∗∗*^*P* < 0.01.

**Table 4:** Performance associated with protein supplement usage.

	Nonsupplement users		*P* value	Protein supplement users		*P* value
	Week 0	Week 8		Week 0	Week 8	
*Exercises*						
1RM back squat (kg)	96.25 (±51.70)	101.1 (±52.83)	^*∗∗∗∗*^	118.2 (±50.60)	122.1 (±50.22)	^*∗∗∗∗*^
1RM bench press (kg)	60.89 (±38.03)	63.99 (±38.26)		80.46 (±36.67)	82.45 (±36.67)	
1RM deadlift (kg)	115.2 (±62.02)	121.7 (±61.79)		142.3 (±62.96)	147.7 (±62.21)	
Total pull-ups (set)	7.62 (±5.96)	8.49 (±6.28)		9.59 (±0.91)	10.22 (±6.08)	

*Performance improvement from week 0 to week 8*						
1RM back squat	8.76 (±3.10)		^*∗∗∗∗*^ †	4.36 (±0.98)		^*∗∗∗∗*^ †
1RM bench press	8.26 (±2.41)			3.44 (±1.15)		
1RM deadlift	10.76 (±4.27)			5.39 (±1.54)		
Total pull-ups	15.57 (±5.21)			9.90 (±2.52)		

Values are Mean (±SD); ^*∗∗∗∗*^ and † represent *P* < 0.0001 between rows and columns, respectively.

## Data Availability

All data used to support the findings of this study are available from the corresponding author upon request.

## Supplementary Materials

Supplementary Materials Supplementary Table 1: demographics of participants. Demographics and lifestyle characteristics of the participants were separated into two categories as supplement versus nonsupplement users. Supplementary Table 2: demographic and lifestyle characteristics of dietary supplement users. Comparison between the supplement users' category and protein consumption category was assessed to depict whether general supplementation or protein supplementation specifically would result in a significant difference among demographic and lifestyle characteristics that compared protein supplement versus nonprotein supplements users. Supplementary Table 3: nutritional knowledge, information sources, and awareness of health risks reported by dietary supplement users among resistance-trained individuals. The questions investigating nutrition knowledge were based on a previously validated questionnaire used to measure the nutrition knowledge for sport (NSKQ) in athletes. The minimum score that can be obtained was 0, whereas the maximum score that can be obtained was 53 based on the fact that the total number of questions to be answered was 53. Participants were then divided into 3 groups: poor, satisfactory, and high knowledge with an outcome score of less than 20, between 20 and 40, and more than 40, respectively. For further analysis, each category of the questionnaire was computed into different scores as per the total number of questions. Supplementary Figure 1: overall diagram of questions and answers of nutritional knowledge questionnaire of the participant. The figure describes in detail the outcomes of answers for each question of the nutrition-related knowledge using the nutrition for sports knowledge questionnaire (NSKQ). Supplementary Figure 2: individual performance between nonprotein supplement users and protein supplement users. To ease the view of individual performance among all participants, the figure illustrates a scattered plot of individual 1RM back squat (A), 1RM bench press (B), 1RM deadlift (C), and set of pull-ups (D) at week 0 and week 8, for nonprotein and protein supplement users.
