# Supplementary material for: Protein Supplement Perceptions, Use, and Associated Performance in Young Lebanese Resistance-Training Athletes
Source: J Nutr Metab. 2022 Feb 18;2022:4150620. doi: 10.1155/2022/4150620 (PMC8881173; doi:10.1155/2022/4150620)

Supplementary material

Supplementary Table 1. Demographics of participants

	Non-Supplement Users n (%)	Supplement Users n (%)	*P* value
Gender			
Male	21 (33.3)	42 (66.7)	0.21
Female	17 (45.9)	20 (54.1)	
Age Group			
20-29	28 (38.9)	44 (61.1)	0.769
30-40	10 (35.7)	18 (64.3)	
Level of Education			
Brevet	1 (33.3)	2 (66.7)	0.224
Lebanese Bachelor	1 (33.3)	2 (66.7)	
Technical Degree	0 (0.0)	4 (100.0)	
Bachelor	25 (47.2)	28 (52.8)	
Masters or Above	11 (29.7)	26 (70.3)	
Others	0 (0.0)	0 (0.0)	
Disease History			
None	34 (36.4)	55 (61.8)	1
Yes	4 (45.5)	7 (63.6)	
Smoking status			
Yes	11 (39.3)	17 (60.7)	0.869
No	27 (37.5)	45 (62.5)	
Alcohol Intake			
Daily	0 (0.0)	0 (0.0)	0.777
Weekly	11 (32.4)	23 (67.6)	
Monthly	9 (40.9)	13 (66.7)	
Yearly	4 (33.3)	8 (66.7)	
Never	14 (43.8)	18 (56.3)	

P value: Pearson's Chi-square test and Fisher exact test with more than 20% of expected counts less than 5 (disease history, calories consumed daily, and grams of food protein consumed daily)

Supplementary Table 2. Demographic and lifestyle characteristics of dietary supplement users

	NPSU n (%)	PSU n (%)	*P* value
Gender			
Male	28 (54.9)	35 (71.4)	0.087
Female	23 (45.1)	14 (28.6)	
Age Group			
20-29	36 (70.6)	36 (73.5)	0.748
30-40	15 (29.4)	13 (26.5)	
Level of Education			
Brevet	2 (3.9)	1 (2.0)	0.196
Lebanese Bachelor	2 (3.9)	1 (2.0)	
Technical Degree	0 (0.0)	4 (8.2)	
Bachelor	30 (58.8)	23 (436.9)	
Masters or Above	17 (33.3)	20 (40.8)	
Others	0 (0.0)	0 (0.0)	
Disease History			
None	45 (88.2)	44 (49.4)	0.803
Yes	6 (11.8)	5 (45.5)	
Smoking status			
Yes	14 (27.5)	14 (28.6)	0.901
No	37 (72.5)	35 (71.4)	
Alcohol Intake			
Daily	0 (0.0)	0 (0.0)	0.779
Weekly	18 (35.3)	16 (32.7)	
Monthly	12 (23.5)	10 (20.4)	
Yearly	7 (13.7)	5 (10.2)	
Never	14 (27.5)	18 (36.7)	
Type of diet			
Balanced	41 (80.4)	40 (81.6)	0.67
Vegetarian	1 (2.0)	1 (2.0)	
Vegan	1 (2.0)	0 (0.0)	
Ketogenic	0 (0.0)	2 (4.1)	
Carb Cycling	2 (3.9)	1 (2.0)	
Flexible Dieting	2 (3.9)	3 (6.1)	
Low Carb	4 (7.8)	1 (2.0)	
Pescatarian	0 (0.0)	1 (2.0)	
Calories Consumed daily			
I don't Know	15 (29.4)	14 (28.6)	0.087
<1000	1 (2.0)	1 (2.0)	
1001-1500	11 (21.6)	2 (4.1)	
1501-2000	14 (27.5)	15 (30.6)	
2001-2500	4 (7.8)	8 (16.3)	
2501-3000	3 (5.9)	6 (12.2)	
3001-3500	1 (2.0)	3 (6.1)	
3501-4000	2 (3.9)	0 (0.0)	
Grams of food protein consumed daily			
I don't Know	30 (58.8)	18 (36.7)	0.136
<50	1 (2.0)	0 (0.0)	
51-100	7 (13.7)	10 (20.4)	
101-150	8 (15.7)	14 (28.6)	
151-200	4 (7.8)	6 (12.2)	
201-250	1 (2.0)	0 (0.0)	
>250	0 (0.0)	1 (2.0)	

P value: Pearson's Chi-square test and Fisher exact test with more than 20% of expected counts less than 5 (level of education, calories consumed daily, and grams of food protein consumed daily).

Supplemental Table 3. Nutritional knowledge, information sources, and awareness of health risk reported by dietary supplement users among resistance trained individuals

	NPSU n (%)	PSU n (%)	*P* value
Knowledge score			
Poor: < 20	6 (11.8)	6 (12.2)	
Satisfactory: 20-39	43 (84.3)	39 (79.6)	0.733
High: ≥ 40	2 (3.9)	4 (8.2)	
Nutritional Information sources			
Internet	28 (54.9)	18 (36.7)	
Social media	4 (7.8)	3 (6.1)	
Sport coach	13 (25.5)	20 (40.8)	
Dietitian	4 (7.8)	4 (8.2)	0.312
Shop representative	1 (2.0)	2 (4.1)	
Peers	1 (2.0)	0 (0.0)	
Family	0 (0.0)	2 (4.1)	
Awareness of Health Risks			
None	24 (47.1)	17 (34.7)	
Excess Calcium Loss	2 (3.9)	3 (6.1)	
Harm Kidney Function	23 (45.1)	26 (53.1)	0.666
Heavy Metal Contamination	0 (0.0)	1 (2.0)	
Other	2 (3.9)	2 (4.1)	

P value: Fisher exact test

Supplementary Figure 1 : Overall diagram of questions and answers of nutritional knowledge questionnaire of the participant

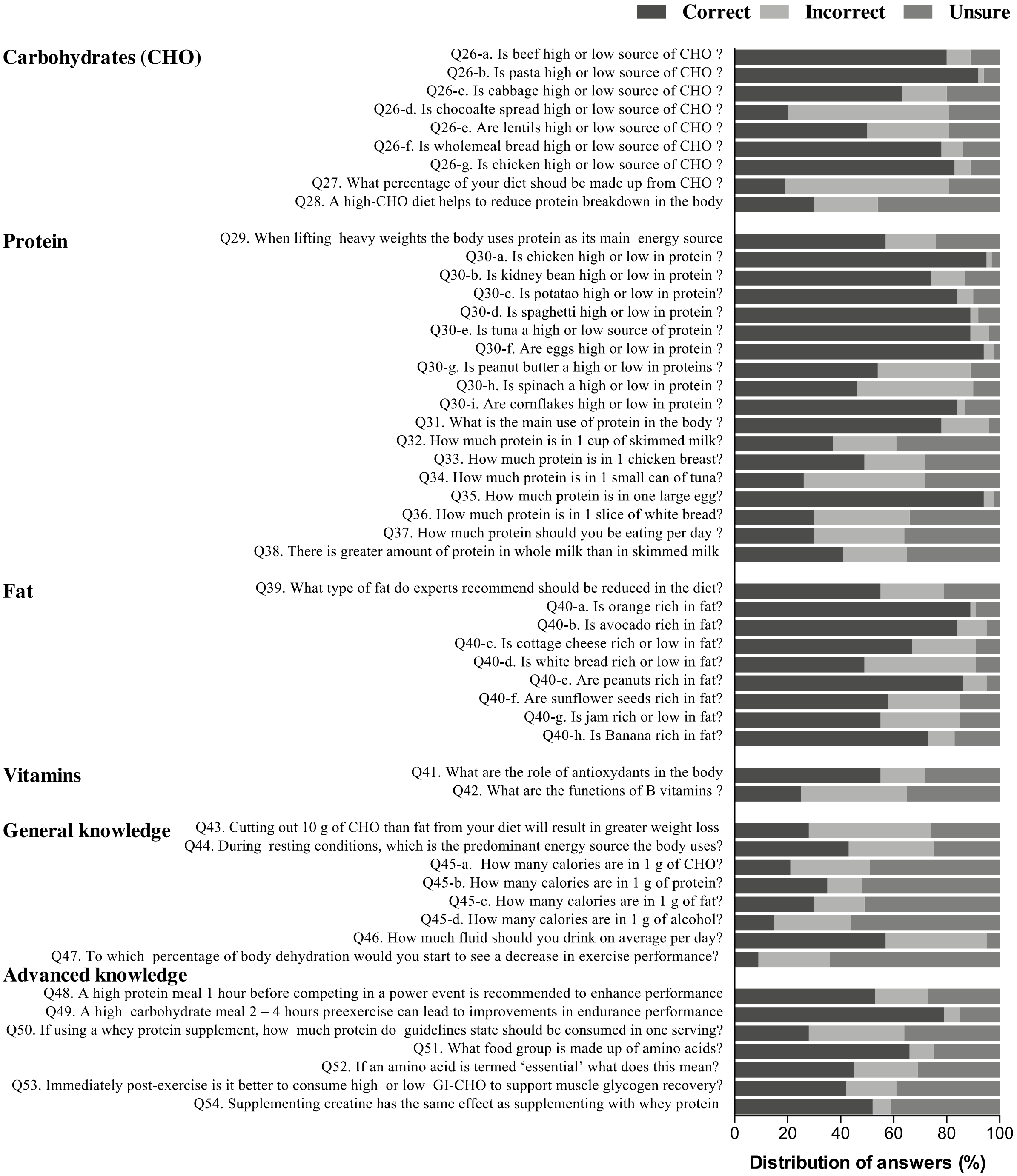

Supplementary Figure 2. Individual performance between non-protein supplement usersa nd protein supplement users.

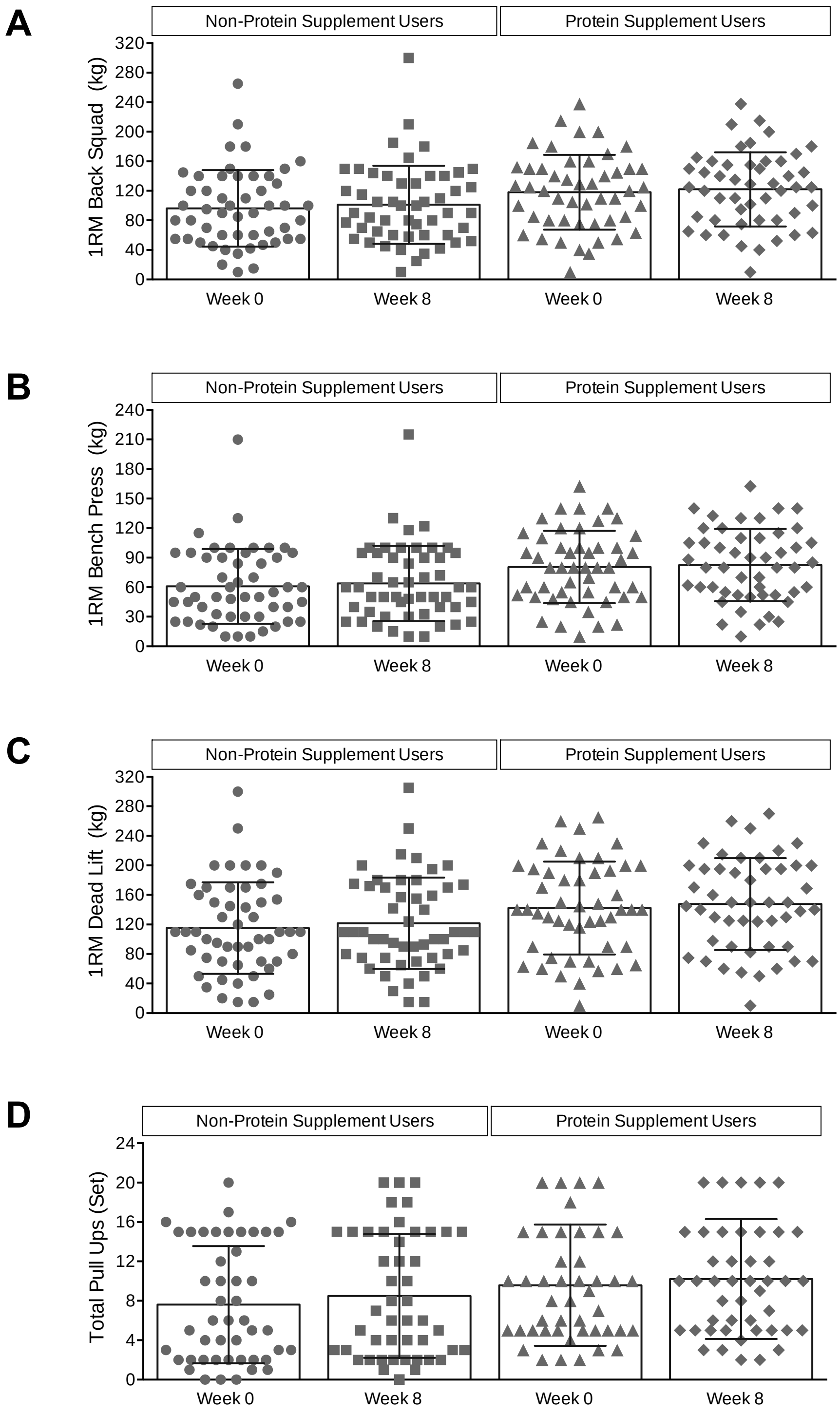

Supplement: Supplementary Materials — Supplementary Table 1: demographics of participants. Demographics and lifestyle characteristics of the participants were separated into two categories as supplement versus nonsupplement users. Supplementary Table 2: demographic and lifestyle characteristics of dietary supplement users. Comparison between the supplement users' category and protein consumption category was assessed to depict whether general supplementation or protein supplementation specifically would result in a significant difference among demographic and lifestyle characteristics that compared protein supplement versus nonprotein supplements users. Supplementary Table 3: nutritional knowledge, information sources, and awareness of health risks reported by dietary supplement users among resistance-trained individuals. The questions investigating nutrition knowledge were based on a previously validated questionnaire used to measure the nutrition knowledge for sport (NSKQ) in athletes. The minimum score that can be obtained was 0, whereas the maximum score that can be obtained was 53 based on the fact that the total number of questions to be answered was 53. Participants were then divided into 3 groups: poor, satisfactory, and high knowledge with an outcome score of less than 20, between 20 and 40, and more than 40, respectively. For further analysis, each category of the questionnaire was computed into different scores as per the total number of questions. Supplementary Figure 1: overall diagram of questions and answers of nutritional knowledge questionnaire of the participant. The figure describes in detail the outcomes of answers for each question of the nutrition-related knowledge using the nutrition for sports knowledge questionnaire (NSKQ). Supplementary Figure 2: individual performance between nonprotein supplement users and protein supplement users. To ease the view of individual performance among all participants, the figure illustrates a scattered plot of individual 1RM back squat (A), 1RM b [file 4150620.f1.pdf]
